# Polymorphisms in MGP gene and their association with lead toxicity

**DOI:** 10.1080/15376510802488181

**Published:** 2009-06-30

**Authors:** Abjal Pasha Shaik, Kaiser Jamil

**Affiliations:** 1 Indo-American Cancer Hospital and Research Centre, Hyderabad, India; 2 Bhagwan Mahavir Medical Research Centre, Hyderabad, India

**Keywords:** Lead toxicity, Blood lead levels, MGP gene polymorphisms, PCR

## Abstract

Matrix γ-carboxy glutamic acid protein (MGP) is a 10-kDa secreted protein containing five residues of the vitamin K-dependent calcium binding amino acid γ-carboxyglutamic acid (Gla). This study was carried out to examine the effects of MGP gene promoter polymorphism (T-138C) on blood lead levels (BLL) and hematological parameters in 113 battery manufacturing unit workers occupationally exposed to lead and 102 controls. Genotypes for the MGP T-138C polymorphism were determined by PCR and restriction fragment length digestion. BLL were determined by Anode Stripping Voltammetry using ESA Model 3010B Lead analyzer. Complete blood picture (CBP) was analyzed using ADVIA Cell counter for each sample. The frequencies of MGP–TT, CT and CC genotypes in our population were 38.6%, 44.3%, and 17.2%, respectively. The frequencies for T and C alleles were 0.612 and 0.386, respectively. Although BLL did not differ significantly among genotypes; they were higher in workers with TT/CT genotype compared to CC genotype subjects (76–88 μg/dL vs 22–45 μg/dL, p > 0.05). About 29.2% of volunteers (n = 33) from the occupationally exposed group had hemoglobin levels below 10.0 gms/dl. There was no significant difference in total white cell count and platelet count between occupational and non-exposed groups. The possible role of SNPs in the promoter region of MGP gene with relation to lead toxicity was investigated for the first time in the Indian population; although significance could not be achieved in this study, further assessments over a larger population size may help in better understanding of the consequences of lead exposure.

## Introduction

Human MGP gene is located at 12p13.1–p12.3 and consists of four exons which codes for a 10-kDa Matrix Gla (γ-carboxyglutamic acid) protein, containing five residues of the vitamin K-dependent calcium binding amino acid γ-carboxyglutamic acid (Gla). (Price et al. 1983, 2000; Price and Williamson 1985; Shanahan et al. 1998; Afshin et al. 2001). MGP was first isolated from the matrix of bone, but is now known to be synthesized in all tissues tested, with the highest levels of synthesis in heart, kidney, lung, and cartilage (Luo et al. 1997; Price et al. 2000). MGP is a member of the family of extracellular mineral-binding Gla proteins, with high accumulation in bone and cartilage (Paul et al. 2002). All currently available scientific evidence indicates that MGP plays a significant role as an inhibitor of mineralization (Price et al. 1983, 2000; Shanahan et al. 1998; Afshin et al. 2001). The accumulation of MGP protein in mineralized bone and calcified cartilage could be due to its calcium binding properties related to the presence of carboxylated residues and greatly exceeds that of the soft tissues, which fail to accumulate MGP under normal conditions but can do so when subjected to abnormal mineralization (Price et al. 2000; Afshin et al. 2001).

Cancela et al. (1990) cloned a human genomic library using an MGP cDNA probe and found that the gene spans 3.9 kb. Each exon of MGP corresponds to the domains found in all known vitamin K dependent vertebrate proteins: a transmembrane signal peptide, followed by putative gamma-carboxylation recognition site, and a Gla-containing domain (Luo et al. 1997; Afshin et al. 2001). MGP contains a fourth exon of unknown function that codes for 11 residues and lies between the transmembrane signal peptide and the putative recognition site for the gamma-carboxylase (Price et al. 2000; Afshin et al. 2001). This 4-exon organization is essentially identical to that of bone Gla protein and is quite different from the 2-exon organization encoding this region in the other known vitamin K-dependent proteins.

Due to the numerous molecular and biochemical effects of lead in adults, children, and infants, understanding the mechanisms of lead toxicity still remains as a major challenge to researchers (Ellenhorn 1997; ATSDR 1998; Bergdahl 1998). Lead exposure has been associated with increased risk of lung, stomach, and bladder cancer in diverse human populations (Fu and Boffetta 1995; Steenland and Boffetta 2000; NTP 2003). Studies have demonstrated lead induced genotoxicity in vitro using cultured human peripheral blood lymphocytes (Shaik et al. 2006).

MGP gene suppresses calcium ion function in the cartilage, and other soft tissues, in addition, lead and calcium are divalent cations, having the same absorption pathways (Goyer 2001). Therefore, Pb^2+^ ions can compete with Ca^2+^; the influence of MGP polymorphism with respect to lead deposition assumes importance in understanding the molecular basis of lead toxicity. The main objective of this study was, thus, to explore the relationship between polymorphisms in the promoter region of MGP gene and investigate its probable association with lead poisoning. The study also assesses the influence of MGP genotypes in the study group with relation to certain hematological parameters like CBP and BLL.

## Materials and methods

### Sample collection and preparation

Two hundred and fifteen volunteers (total 198 men and 27 women) aged from 18 to 51 years living in the city of Hyderabad (Andhra Pradesh, India) were enrolled in this study. One hundred and thirteen subjects were working occupationally in the lead battery industry. Only subjects reporting at least two of the symptoms of lead toxicity like headache, nausea, gastritis, vomiting, lethargy, and poor appetite were enrolled in the study. One hundred and two volunteers not-exposed occupationally to lead formed the controls. This study was approved by the Institutional Review Board and Ethics Committee, and each subject provided written informed consent. Details of previous medical history, present health status, nutritional status, years of exposure, and duration of working hours were recorded. Five milliliters of venous blood samples were collected from each volunteer in two tubes, one with heparin for metal analysis, and one containing EDTA for hematological evaluations.

### Determining hematological parameters

Complete blood picture (CBP) was determined using ADVIA Cell counter for each sample. This included Hemoglobin, platelet count, total WBC count, Total RBC count, PCV, and MCV.

### Blood lead level estimation

For the estimation of BLL, ESA Model 3010B Lead analyzer was used, which determines the level of lead in blood by anode stripping voltammetry (ASV). The fundamental principle of operation is when a sufficiently large negative potential is placed on an electrode, lead ions in solution are plated on a mercury electrode. After a fixed plating time, the potential is made less negative to strip the metal off the electrode. The observed current during the stripping process is integrated, and is directly proportional to the concentration of the lead in the sample. The concentrations of the calibrators provided by ESA are determined with reference to standards from NIST and CDC. All the precautions were taken while sampling and processing to avoid lead contamination. Estimations of BLLs were performed at Secunderabad Diagnostic Center (Hyderabad, A.P), which is accredited by the National Accreditation Board for Testing and Calibration Laboratories (NABL) for following ISO/IEC 17025 Standards.

### Genotype determination for the MGP gene polymorphism

Polymerase chain reaction (PCR) is a technique widely used in molecular biology to amplify a piece of DNA by in vitro enzymatic replication using stable DNA polymerase. As PCR progresses, the DNA template is exponentially amplified across several orders of magnitude, generating multiple copies of the DNA fragment of interest. An assay based on polymerase chain reaction (PCR)–Restriction fragment length polymorphism (RFLP) was used to determine the genotype of the MGP gene, as described previously (Zhang et al. 2003). PCR was performed in a 50 μl reaction volume containing 0.5 μM of each primer was used, as below.

- Sense primer: 5′-AAGCATACGATGGCCAAAACTTCTGCA-3′
- Antisense primer: 5′-GAACTAGCATTGGAACTTTTCCCAACC-3′

The amplification reaction was done using 200 μM of each dNTP, 10X PCR buffer supplied by Bangalore Genei, 2.5 mM MgCl_2_, and 3U Taq DNA polymerase. The running conditions were pre-denaturation at 94°C for 5 minutes, followed by 35 cycles of denaturation at 94°C for 30 seconds, annealing at 60°C for 30 seconds, and synthesis at 72°C for 1 minute. Final extension was conducted at 72°C for 5 minutes. The amplified products were digested overnight with BsrI at 65°C and the fragments were separated by electrophoresis in 12% polyacrylamide gel, and visualized by silver staining. The TT genotype is characterized by a 118bp band, CT genotype (118 & 142 bp bands) and CC genotype (142 bp band).

### Statistical analyses for polymorphism detection

Chi-square analysis was used to determine whether the genotype distribution was in Hardy-Weinberg equilibrium and to compare distributions of alleles and genotypes in the different groups of subjects (Zar 1996). *P*-value was analyzed for the significance. Statistical analyses were performed with the MedCalc statistical program.

## Results

Table 1 summarizes the basic characteristics of the study subjects. During the time of presentation, 12 of the 113 occupationally exposed volunteers reporting symptoms of lead toxicity appeared severely malnourished. Blood samples collected from all volunteers were analyzed for CBP and only the parameters related to anemic conditions like hemoglobin are presented here. This is because the hemoglobin levels showed a very wide range of variation, as represented in Table 2. Hemoglobin levels of occupational workers ranged between 7.0–14.0 gms/dl, whereas Hb levels in unexposed workers was 11.3–14.8 gms/dl. It was observed that 33 subjects from the exposed group had low Hb levels (below 10 gms/dl) compared to the controls. Packed cell volume (PCV) levels ranged from 28–37%; there was no significant difference in total white cell count and platelet count between occupational and non-exposed groups. The Genotype frequencies of MGP–TT, CT, and CC were 38.6%, 44.3%, and 17.2%, respectively. The distribution of genotypes was in Hardy-Weinberg equilibrium. Similarly, the allele frequencies for T and C alleles were 0.612 and 0.386, respectively. BLL did not differ significantly among genotypes; however, subjects with TT+CT genotypes showed higher BLL than those with mutated homozygotes CC (76–88 μg/dL vs 22–45 μg/dL, *p* > 0.05). Blood lead levels of occupationally exposed individuals were much higher compared to the non-exposed group (Table 3). Twelve of 33 exposed subjects were malnourished, and severely anemic with hemoglobin levels below 10 gmd/dl. These data indicate that of the 113 occupationally exposed individuals, the MGP CC subjects (*n* = 17) showed a hemoglobin range of 7.0–13.4 gms and a BLL of up to 88 μg/dl. The MGP TT/CT individuals (*n* = 96) showed a hemoglobin range of 10.1–14.2 gm% and a BLL within a range of 22–45 μg/dl. In the control group, the MGP CC (*n* = 20) subjects showed a BLL of 3.4 μg/dl and hemoglobin level up to 14.8 gms/dl, respectively. The MGP TT/CT individuals (*n* = 82) showed a hemoglobin of 11.2–14.0 gms/dL and a BLL of 2 μg/dl). There was no significant difference in total white cell count and platelet count between occupational and non-exposed lead-exposed groups.

**Table 1:** Characteristics of the study group (n = 225).

Parameters	Range of exposed	Non-exposed
Age range	18–51 years	18–51 years
Sex	100 men and 13 women (total 113)	98 men and 14 women (total 102)
Occupational exposure period	4–10 years	Not exposed
Alcohol consumption	31 frequently and 8 occasionally	nil
Smoking and tobacco chewing	40	9
Medical history	Most of them complained about symptoms like gastritis and headache	nil

**Table 2:** Hemoglobin ranges of the study group (occupationally exposed and controls) along with normal ranges.

		No. of subjects	
		
S.No	Range of Hb (gm/dl)	Exposed	Control
1	7.0–8.0	5	—
2	8.1–9.0	9	—
3	9.1–10.0	19	—
4	10.1–11.0	39	—
5	11.1–12.0	22	44
6	12.1–13.0	13	37
7	13.1–14.0	6	21
Total		113	102

Number of highly anemic individuals in the exposed group was 33. Twelve malnourished volunteers were among these 33 highly anemic individuals.

Normal ranges: Hb: Males 13.5–18.0 gms/dl, Females 11.5–16.5 gms/dl; PCV: Males 40–54%, Females 37–47%; WBC: 4,000–11,000 cells/mcl; Platelets: 1.5–5 lakhs/cumm.

**Table 3:** Variations of blood lead levels and hemoglobin levels in MGP TT/CT and CC individuals.

S.No	Subjects	Polymorphism	Hb (gm%)	BLL (μg/dl)
1	Occupationally exposed individuals (*n* = 113)	MGP (TT/CT) (*n* = 96)	10.1–14.2	76–88
		MGP (CC) (*n* = 17)	7.0–13.4	21.8–45
2	Control group (*n* = 102)	MGP (TT/CT) (*n* = 82)	11.2–14.0	0.6–3.4
		MGP (CC) (*n* = 20)	11.3–14.8	1.0–1.9

*p* > 0.05.

## Discussion

Genetic association studies with candidate genes are widely used to study complex diseases caused by epigenetic factors (Cardon and Bell 2001). This study aims to identify the relation of some SNPs with emphasis on lead toxicity. Since MGP gene is an important biomarker associated with atherosclerotic calcification and involved in calcium metabolism; it can be hypothesized that it may also be associated with lead toxicity. Therefore, this study tries to correlate the influence of MGP promoter gene polymorphisms with BLL in a group of battery manufacturers who are occupationally exposed to lead. Blood lead level (μg/dl) is the biologic index most often used by health care providers as an indicator of recent lead exposure (Astrin et al. 1987; Bressler and Goldstein 1991). As lead interrupts the heme synthesis it was essential to investigate the hematological parameters (CBP) of all individuals (both exposed and controls).

Studies by Paul et al. (2002) on rats to evaluate the function of MGP in bone metabolism indicate an elevation in serum levels of MGP after etidronate injections and its strong binding to bone mineral, thus inhibiting abnormal calcification of arteries and other soft tissues. Recent genetic and biochemical studies have established MGP as the first protein known to act as a calcification inhibitor in vivo (Speer and Giachelli 2004). The ability of MGP to bind with great avidity to the mineral complex suggests that MGP is a strong inhibitor of crystal growth. Afshin et al. (2001) discovered that MGP gene starts with the T-138C polymorphism which influences gene expression level; CC genotype MGP showing the highest levels in blood serum followed by CT and TT. The C genotype (CT+CC) tended to show a higher calcification factor than the TT genotype. Extrapolating from these studies indicate that MGP might form soluble complexes with the metallic ions, which may be discharged easily by the biological system in vitro. Compared with the CC genotype, the TT genotype does not favor the lead to discharge, causing the storing up of lead. Therefore, BLL of TT genotype was found to be higher than the CC genotype. Presence of polymorphism in MGP gene may alter the levels of lead in the skeleton and soft tissue which causes storing up of lead in reverse proportion to MGP level. Considering the central role of MGP in vascular calcification and a similar pathogenesis between vascular calcification and kidney stones, we hypothesized that MGP genetic polymorphisms may influence the risk of lead toxicity.

To our knowledge, this is the first study describing significant effects of the MGP T-138C polymorphism on BLL and hematological parameters in battery-exposed workers form Hyderabad (A.P., India). The frequency of MGP CC genotype was low in our study group. Previously, an investigation was performed in a Chinese Han population in children by Zhang et al. (2003), who showed significant effect of MGP gene polymorphisms with BLL.

Further studies are necessary in larger population samples to demonstrate the exact involvement of MGP gene in the etiology of lead poisoning. In addition to MGP, polymorphisms in many other genes like ALAD, VDR, PBG Synthase, etc., may affect the susceptibility to lead exposure. Rezende et al. (2008) showed lower plasma lead, blood lead, and their ratio in subjects with haplotype polymorphisms, suggesting that VDR haplotypes modulate the circulating levels of lead in exposed subjects. The role of hemochromatosis gene in the absorption of lead and symptoms of lead toxicity are also being increasingly examined (Onalaja and Claudio 2000). In our analysis of T-138C MGP polymorphism among worker individuals with TT/CT genotypes showed higher BLL (above 70 μg/dl). Polymorphism of T-138C may have influenced the promoter region leading to altered protein function during transcription (Afshin et al. 2001). The highest BLLs in our sub-population were seen in TT genotype individuals followed by CT and CC genotypes. In our study, subjects from the occupational group had lower hemoglobin levels (below 10 gms/dl) than the unexposed group, and there was no significant difference between subtypes of MGP genotype with relation to their hemoglobin levels. There was a decrease in hemoglobin levels of malnourished volunteers who were among the 33 highly anemic individuals. Packed cell volume (PCV) levels ranged from 28–37% which was low compared to normal levels. Improved iron nutrition has been found to be important in the reduction and mitigating the effects of occupational and environmental exposure to lead (Mahaffey 1995; Shaik et al. 2006).

However, in the population that we have studied, although levels of lead were varying with respect to polymorphism, significance could not be achieved. This may be because of the sample size; therefore, higher sample size may be required to establish the role of MGP gene SNPs in lead toxicity. Further studies on clinical implications of such high exposures in both genotype individuals on a larger population size may throw more insight with respect to analyses of lead toxicity.
